# Intravital molecular imaging reveals the restrained capacity of CTLs in the killing of tumor cells in the liver

**DOI:** 10.7150/thno.44979

**Published:** 2021-01-01

**Authors:** Lei Liu, Bolei Dai, Ruixue Li, Zheng Liu, Zhihong Zhang

**Affiliations:** 1 Britton Chance Center for Biomedical Photonics, Wuhan National Laboratory for Optoelectronics-Huazhong University of Science and Technology, Wuhan, Hubei 430074, China.; 2 MoE Key Laboratory for Biomedical Photonics, Collaborative Innovation Center for Biomedical Engineering, School of Engineering Sciences, Huazhong University of Science and Technology, Wuhan, Hubei 430074, China.

**Keywords:** molecular imaging, liver tumor, CTL, apoptosis, calcium signaling

## Abstract

Cytotoxic T lymphocytes (CTLs) and their gene-engineered cells display great application prospects in tumor immunotherapy. The timing of CTL-induced molecular events in tumor cells is unclear, and we also unknow whether the killing efficiency of CTLs is restrained in the liver, an immunotolerant organ with a high tumor incidence.

**Methods:** We used intravital imaging to dynamically monitor the fluorescence resonance energy transfer (FRET) signals of caspase-3 and calcium sensor in tumor cells after transferring CTLs into tumor-bearing mice.

**Results:** Our data show that several CTLs attacked on one tumor cell, and on average each CTL killed 1.24 ± 0.11 tumor cells per day in the liver, which was much less efficient than that in the spleen (3.18 ± 0.26 tumor cells/CTL/day). The killing efficiency of CTLs is restrained in the liver and can be reversed by blocking immunosuppressive cytokine. Tumor cells exposed to CTLs appeared to have prolonged calcium influx, which occurred dozens of minutes before caspase-3 activity.

**Conclusion:** The quantitative characterization of these molecular and cellular events provides accurate information to evaluate the efficiency of cellular immunotherapy against tumors and understand the impact of an organ's immune status.

## Introduction

Cytotoxic T lymphocytes (CTLs) are central effector cells in the adaptive immune system that eliminate tumors 1, 2. CTLs recognize the homologous MHC polypeptide antigen complex on the surface of tumor cells through the T-cell receptor (TCR), come in stable contacts with tumor cells to form an immune synapse, and then secrete IFN-γ, perforin, and granzyme to kill tumor cells 3-5. During this process tumor cells respond to the attack of CTLs and generate calcium influx and apoptosis 6. Cellular immunotherapy based on the adoptive transfer of naturally occurring or gene-engineered T cells to patients has shown attractive promises in different tumors, such as tumor-infiltrating lymphocytes (TILs) in melanoma 7 and chimeric antigen receptor (CAR) T cells in leukemia 8. However, the adoptive cell therapy (ACT) treatment effects on different individuals vary widely. CTLs have strong tumor-killing capabilities *in vitro*, but their effective killing of tumor cells *in vivo* not only required to sense homologous antigens, but they also need to migrate through the vascular barrier into tumor areas, and be regulated by the tumor microenvironment. CTL efficiency in killing tumor cells is related to many factors *in vivo*, such as the level of MHC expression in tumor cells, the activity of CTLs 9, 10, the effect of the target ratio of CTLs and tumors 11, and the tumor microenvironment. It is challenging to simulate these factors with a co-culture of CTLs and tumors *in vitro*. Therefore, establishing a method to characterize the killing capacity of CTLs *in vivo*, especially in the real tumor microenvironment, is of significant necessity.

The liver is the largest internal organ with specific anatomical structure and regional immune properties that normally maintain immunotolerance. It is a high-risk organ of hepatocellular carcinoma and cancer metastasis 12. Cellular immunotherapy has shown promise in liver tumor treatment; it controls the growth of tumors and prolongs the survival rate of tumor-bearing mice and patients, displaying clinical application potential 13-15. The immune cells with an anti-tumor effect in the liver are mainly CTLs 16, NK cells 17, 18, and NKT cells 19. At present, the ACT treatment for liver tumors has not achieved satisfactory outcomes 20, 21. In addition to the unique escape mechanism of tumors, such as the tumor immunosuppressive microenvironment, avoiding immune destruction, and resisting cell death 22-24, the highly immune-tolerant environment in the liver is a great challenge to the development of effective CTL immunotherapies for liver cancer 25. It is still not clear how CTLs exert their anti-tumor effects under the immune-tolerant environment of the liver and if there is a difference of CTL killing efficiency between liver and normal immune status organs.

Optical molecular imaging techniques provide powerful tools to reveal cell movement 26 and function *in vivo*, which can be used to dynamically visualize cell interaction 27-29 and monitor changes in the molecular signals of individual cells 30, 31. Genetically encoded biosensors are the most commonly used probes for intravital molecular imaging. At present, apoptosis, calcium signaling, ERK, and NADH/NAD^+^ signaling are already available for intravital imaging. These biosensors are used to explore tumor cell apoptosis 32, track T lymphocyte function 31, explain the mechanism of tumor cell resistance to chemotherapy 33, and monitor the metabolic status of leukemia-initiating cells* in vivo* 34. As of the moment of writing, intravital molecular imaging of tumor apoptosis and calcium signaling in the liver has not been reported.

Here, to reflect tumor cell apoptosis and the early response of tumor cells to CTLs, caspase-3 activity and calcium influx in tumor cells were characterized using genetically encoded fluorescence resonance energy transfer (FRET) 35 probes, which are composed of the fluorescence protein pair of mCerulean3 and cpVenus. We developed a multicolor fluorescence labeling model to quantitatively evaluate the capacity of CTLs to kill tumors in the liver, that tumor cells stably co-expressed ovalbumin (OVA) protein and FRET probe, and activated OT-I CTLs expressing the tdTomato fluorescent protein were transferred into tumor-bearing mice. Intravital molecular imaging data indicated that tumor cell death usually contributes to the accumulated effect of multiple CTLs in the tumor lesions. Compared with a normal immune organ (spleen) the capacity of CTLs to kill tumor cells is restrained in the liver, with one CTL killing only 1.24 ± 0.11 tumor cells per day. CTLs triggered prolonged calcium influx (> 30 s) in tumor cells, which the trigger time of prolonged calcium influx is around 40 min earlier than the occurrence of caspase-3 activity. These results help to accurately quantify the capability and mechanism of CTLs to kill tumor cells in the liver, and to understand the role of lymphocytes in the treatment of liver tumors.

## Results

### Screening melanoma B16 cells with stable co-expression of the caspase-3 probe and ovalbumin protein

**Figure 1:**
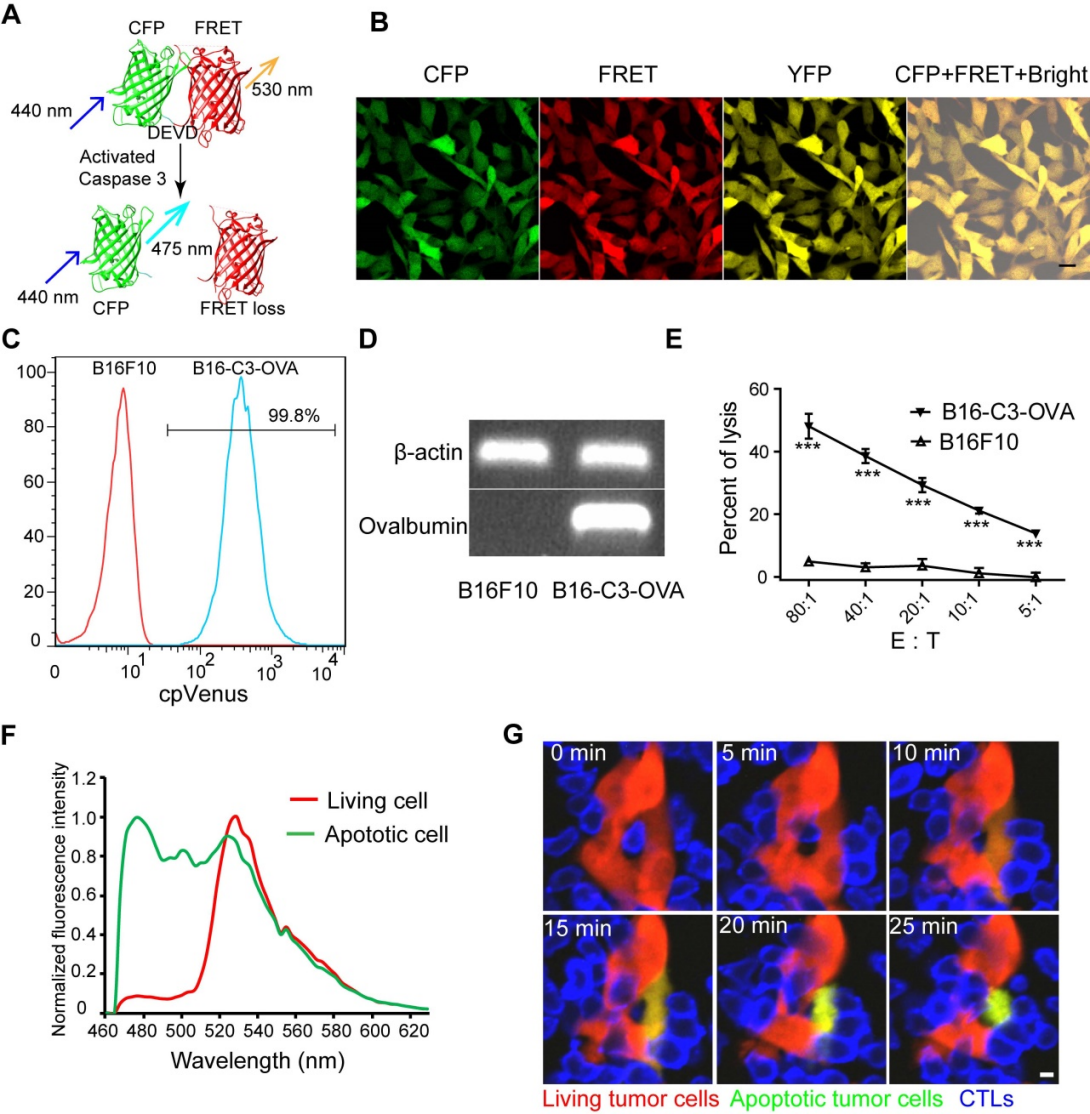
** Activated OT-I CD8^+^ T cells specifically recognize and induce the apoptosis of B16-C3-OVA melanoma cells.** (A) Schematics of the caspase-3 FRET probe. mCerulean3 (CFP) and cpVenus (FRET) fluorescent proteins are linked by the DEVD peptide, which can be cleaved by activated caspase-3. (B) Confocal imaging of B16-C3-OVA cells. Scale bar is 20 µm. (C) Flow cytometry for C3 probe expression in B16-C3-OVA cells. Red histogram, negative control B16F10; blue histogram, B16-C3-OVA. (D) Semi-quantitative PCR to detect the mRNA level of OVA in B16-C3-OVA cells. (E) Flow cytometry (CFSE and propidium iodine-based assays) confirms activated OT-I CD8^+^ T cells killing tumor specificity. Tumor cells were co-cultured with activated OT-I CD8^+^ T cells for 8h to detect tumor cell death. The two-way ANOVA analysis was used for statistical analysis, *** *P* < 0.001. (F) The emission spectrum scanning of living or apoptotic B16-C3 cells. We performed confocal spectral scanning imaging 24 h post adding apoptosis inducers. (G) Dynamic visualization of the FRET signal change during activated OT-I CD8^+^ T cells killing of B16-C3-OVA cells* in vitro*. The CD8^+^ T cells / B16-C3-OVA ratio is 20:1. Scale bar: 5 µm.

To explore the specific killing of tumor cells by CTLs *in vivo*, we choose mCerulean3 and cpVenus fluorescent proteins, variants of cyan and yellow fluorescent proteins 36, 37, as a FRET pair to construct a new genetic encoded probe for detecting caspase-3 activity, denoted as C3. Reportedly, the mCerulean3 and cpVenus pair is an optimal FRET pair with a large dynamic range (~800% in cuvette and ~104% in hippocampal neurons when used as a calcium probe) and bright donor fluorescence from mCerulean3 31. As shown in Figure 1A, mCerulean3 (donor) and cpVenus (acceptor) were linked with the DEVD sequence (the substrate peptide of Caspase-3), which possessed a high FRET efficiency when C3 was intact. C3 lost the FRET signal when the DEVD linker was cleaved by the activated caspase-3, indicating cell apoptosis. The gene sequence of the C3 probe and ovalbumin (OVA) were connected using internal ribosome entry site (IRES) (Figure S1A), leading to each tumor cell simultaneously expressing the C3 probe and OVA protein, which could be recognized specifically by OT-I CTLs and characterize caspase-3 activity.

The B16F10 melanoma cell line has strong liver metastasis properties and is a common model for studying tumor immunity 27, 38. We used it to screen a cell line, denoted as B16-C3-OVA that co-expressed the C3 probe and OVA protein. We also screened another B16F10 melanoma cell line, denoted as B16-C3, which only expressed the C3 probe. Both confocal imaging and flow cytometry confirmed that almost all B16-C3-OVA cells stably expressed the C3 probe (Figure 1B-C), and semi-quantitative PCR revealed that the OVA protein was expressed in B16-C3-OVA cells (Figure 1D). *In vitro* killing experiments verified that activated OT-I CTLs killed OVA positive tumor cells (B16-C3-OVA) specifically and posed no danger to OVA negative cells (B16F10) (Figure 1E).

We next used apoptosis inducers (Apopisa and Apobid, 1: 1000; Beyotime) to induce cell apoptosis *in vitro*, and performed confocal spectral scanning imaging 24 h post administration. Our data showed that the FRET signal in partial tumor cells was significantly lost, indicating that the C3 probe is sensitive to showing the apoptosis of tumor cells (Figure 1F). It's worth noting that the dynamic range of C3 at the cellular level is enormous (~1189%). By using time-lapse confocal imaging, we dynamically observed the whole process of tdTomato-expressed activated OT-I CD8^+^ T cells recognizing B16-C3-OVA cells and inducing their apoptosis *in vitro* (Figure 1G and Video 1). Because most of the activated OT-I CD8^+^ T cells expressed granzyme B (Figure S1B), they were considered as OT-1 CTLs. Using B16-C3-OVA cells and OT-I-tdTomato CTLs, we established a multicolor model suitable for intravital optical imaging of the tumor microenvironment in the liver, an organ with strong autofluorescence interference.

### The adoptive transfer of OT-I CTLs specifically inhibits the liver metastasis of OVA-expressing tumors

**Figure 2:**
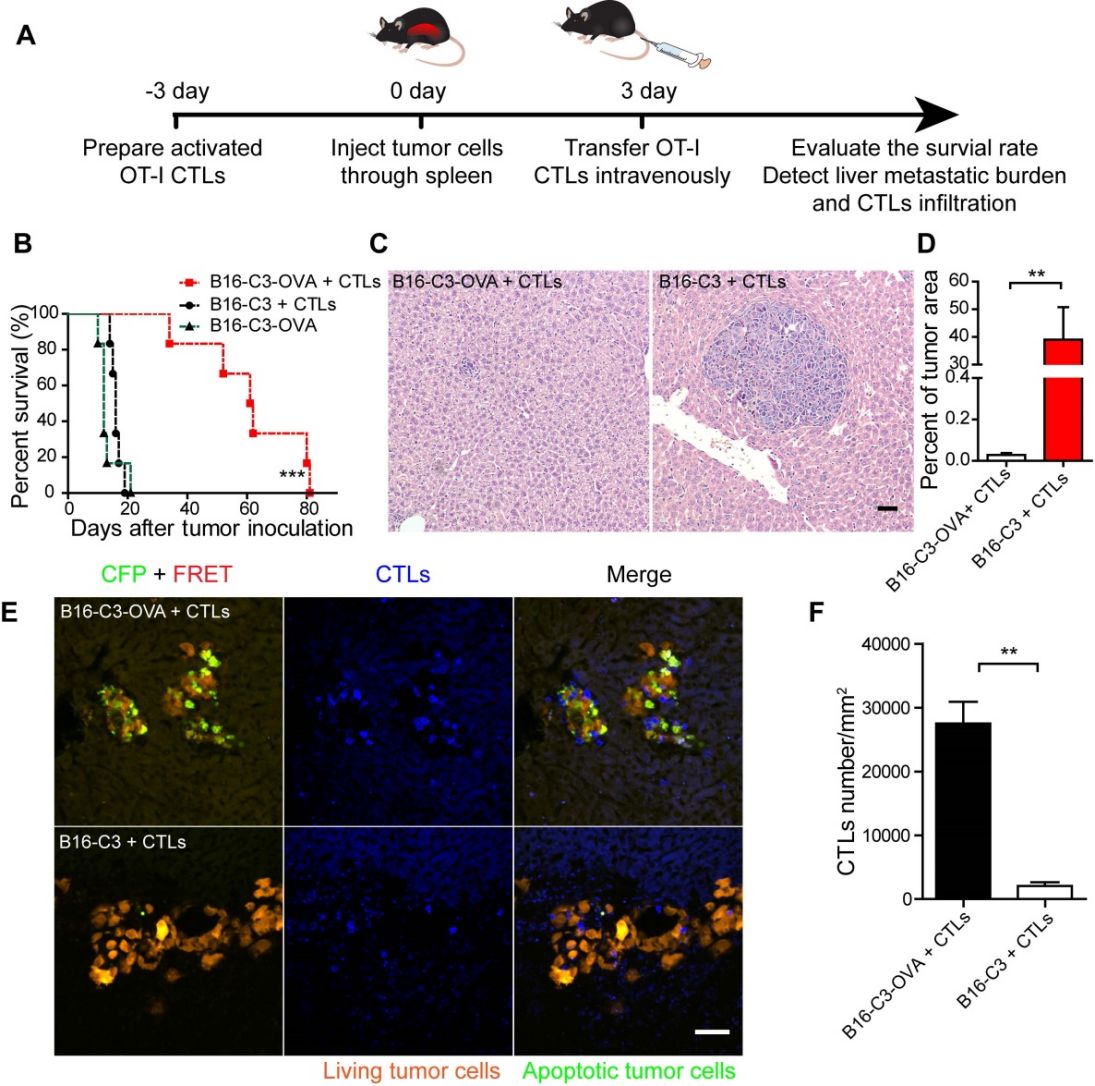
** The adoptive transfer of activated OT-I CD8^+^ T cells efficiently controls the liver metastasis of OVA-expressing tumors.** (A) The schedules of the adoptive cell therapy for the liver metastasis model, in which tumor cells were inoculated into the liver through the spleen. Three days later, the mice were transferred with 2.5×10^6^ activated OT-I CD8^+^ T cells, and the therapeutic effect was assessed. (B) Survival curves of mice with B16-C3 and B16-C3-OVA liver metastases. C57BL/6 mice were adoptively transferred with or without activated OT-I CD8^+^ T cells, n = 6 mice per group. The log-rank test was used for statistical analysis, *** *P* < 0.001. (C) Representative images of hematoxylin and eosin (H&E) staining of liver sections from liver metastasis mice on day 9; mice were transferred with activated OT-I CD8^+^ T cells. Scale bar: 50 µm. (D) The percentages of the tumor metastasis areas in the liver lobe. Each group data were pooled from 7 mice. The unpaired t-test was used for statistical analysis, ** *P* < 0.01. (E) Representative cryosection images of CTLs infiltrating the liver metastases lesions. Scale bar: 50 µm. (F) Number of CTLs per square millimeter in the tumor areas. Data were pooled from 6 mice. The unpaired t-test was used for statistical analysis, ** *P* < 0.01.

Having obtained a B16-C3-OVA cell line that can be recognized specifically and killed by OT-I CTLs, we investigated whether the adoptive transfer of activated OT-I CTLs could inhibit the generation of B16-C3-OVA liver metastasis* in vivo*. We prepared a liver metastasis model by injecting 5 × 10^5^ tumor cells into the hemispleen, followed by the intravenous inoculation of 2.5 × 10^6^ activated OT-I CTLs into mice 3 days later (Figure 2A). The survival rate of B16-C3-OVA liver metastasis mice was enhanced on average to 61 days after the adoptive transfer of OT-I CTLs (Figure 2B). Mice inoculated with B16-C3 tumor cells and adoptive OT-I CTLs, and mice only inoculated with B16-C3-OVA tumor cells all died by days 19 and 21, respectively.

We quantified the liver metastatic burden 4 and 9 days after tumor inoculation by hematoxylin and eosin (H&E) stained liver tissue sections. The results showed that the percent of melanoma colonies area is no significant difference between B16-C3-OVA + CTLs group and B16-C3 + CTLs group on day 4 (Figure S2A-B). On day 9, adoptive transfer of OT-I CTLs inhibited the growth of B16-C3-OVA liver metastasis, with the mean percentage of the tumor area in the liver section only 0.03 ± 0.01%, which was much less than that of the B16-C3 + CTLs group, with 38.99 ± 11.8% (Figure 2C-D and S2C).

To make sure the generation of liver metastasis and adoptive OT-I CTLs infiltration into tumor regions, we performed a cryosection of the liver one day after CTL transfer (4 days after tumor inoculation). As the fluorescent imaging shows in Figure 2E, many CTLs expressing the tdTomato fluorescent protein, infiltrated the metastasis lesions, and a substantial number of apoptotic cells with activated caspase-3 (lost FRET signal and shown as green color) was observed in the B16-C3-OVA group after 1 day of CTL transfer. As control most of the B16-C3 cells were alive (strong FRET signal and shown as red color). The number of adoptive CTLs in the B16-C3-OVA group was significantly higher than that in the B16-C3 group (mean 27524/mm^2^ *vs* 2058/mm^2^, Figure 2F). These results indicate that adoptive CTLs were highly effective in infiltrating the liver metastasis lesions and induced the apoptosis of OVA positive tumor cells, providing key factors for the inhibition of the growth of the liver metastasis and extending the survival time of tumor-bearing mice.

### Intravital imaging reveals the motility properties of CTLs in liver metastasis lesions

**Figure 3:**
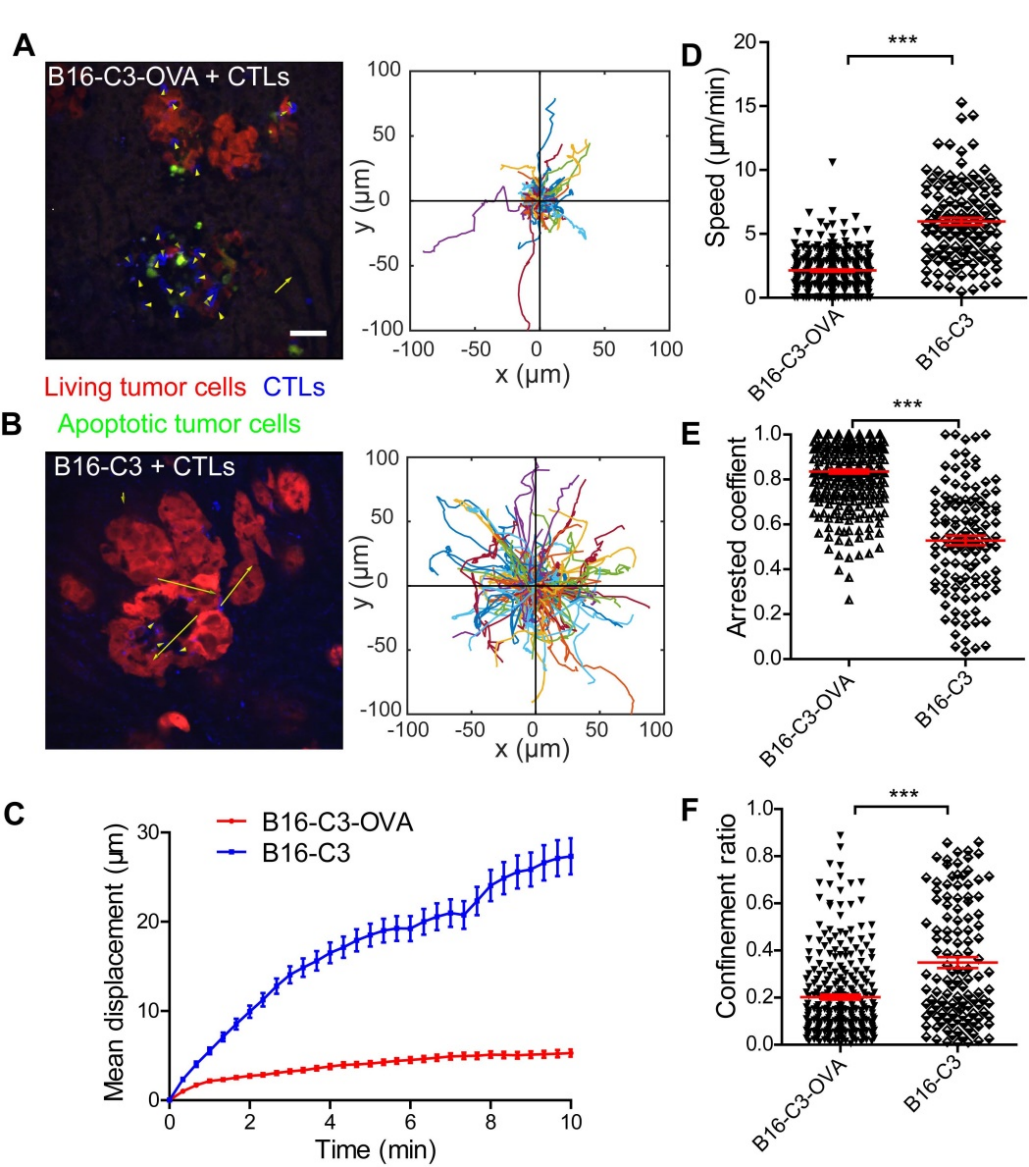
** The motility properties of CTLs in liver metastasis.** (A and B) A typical image of CTLs displacements and tumor metastasis lesions in the liver. The scale bar is 40 µm, and arrows represent CTLs displacement. The trajectories of CTLs movements were tracked and extracted following the alignment of their starting positions. Data were pooled from 5-7 mice in each group from three independent experiments. (C) Mean displacement of CTLs tracked for 10 mins. (D-F) Statistical analysis of (D) the mean speed, (E) the arrest coefficient, and (F) the confinement ratio. Each point represents one CTL from a 30-min video; red bars represent the mean ± SEM. Data were pooled from 5-7 mice in each group from three independent experiments. The unpaired *t*-test was used for statistical analysis, *** *P* < 0.001.

Cell movement is closely related to its function 39. To visualize the movement behavior of CTLs in the liver and confirm the contact between CTLs and metastatic tumor cells, we prepared a liver metastasis model as shown by the method in Figure 2A for intravital imaging. According to the cryosection data on CTLs infiltration shown in Figure 2E, we chose one day after OT-I CTL transfer to take time-lapse intravital imaging of the liver metastasis microenvironment, in which CTLs expressed the tdTomato fluorescent protein (shown in blue) and B16 melanoma cells expressed the C3 probe (shown in red [alive] or green [apoptosis]). The duration of intravital imaging was 30 min. Compared with the B16-C3 group, most CTLs in the B16-C3-OVA group were in stable contact or surround tumor cells, which the trajectories of most CTLs in the B16-C3-OVA group were limited in a very small range (Figure 3A-B and Video 2-3). The motility of CTLs in the B16-C3-OVA group reduced significantly, the mean displacement of CTLs in the B16-C3-OVA group in 10 minutes was shorter than that in the B16-C3 group (5.3 ± 0.4 μm *vs* 27.3 ± 2.0 μm, Figure 3C), and CTLs movements in the B16-C3-OVA group were at a much slower speed (2.13 ± 0.09 μm/min *vs* 5.97 ± 0.28 μm/min, Figure 3D). The arrest coefficient that the percentage of cell “resting” time (when the immediate speed is less than 2 μm/min) in the B16-C3-OVA group was higher than that in the B16-C3 group (Figure 3E, percentages of 83.6 ± 0.9 *vs* 52.8 ± 2.1). CTL confinement ratio (the ratio of the displacement to the total length of the path of CTL) in the B16-C3-OVA group was significantly lower than that in the B16-C3 group (0.202 ± 0.010 *vs* 0.348 ± 0.023, Figure 3F). Intravital imaging of the tumor metastasis microenvironment revealed the restricted movement of CTLs in the B16-C3-OVA group, indicating that CTLs recognize and interact with tumor cells to trigger tumor cell death.

### CTLs are lowly efficient in killing tumor cells in the liver

**Figure 4:**
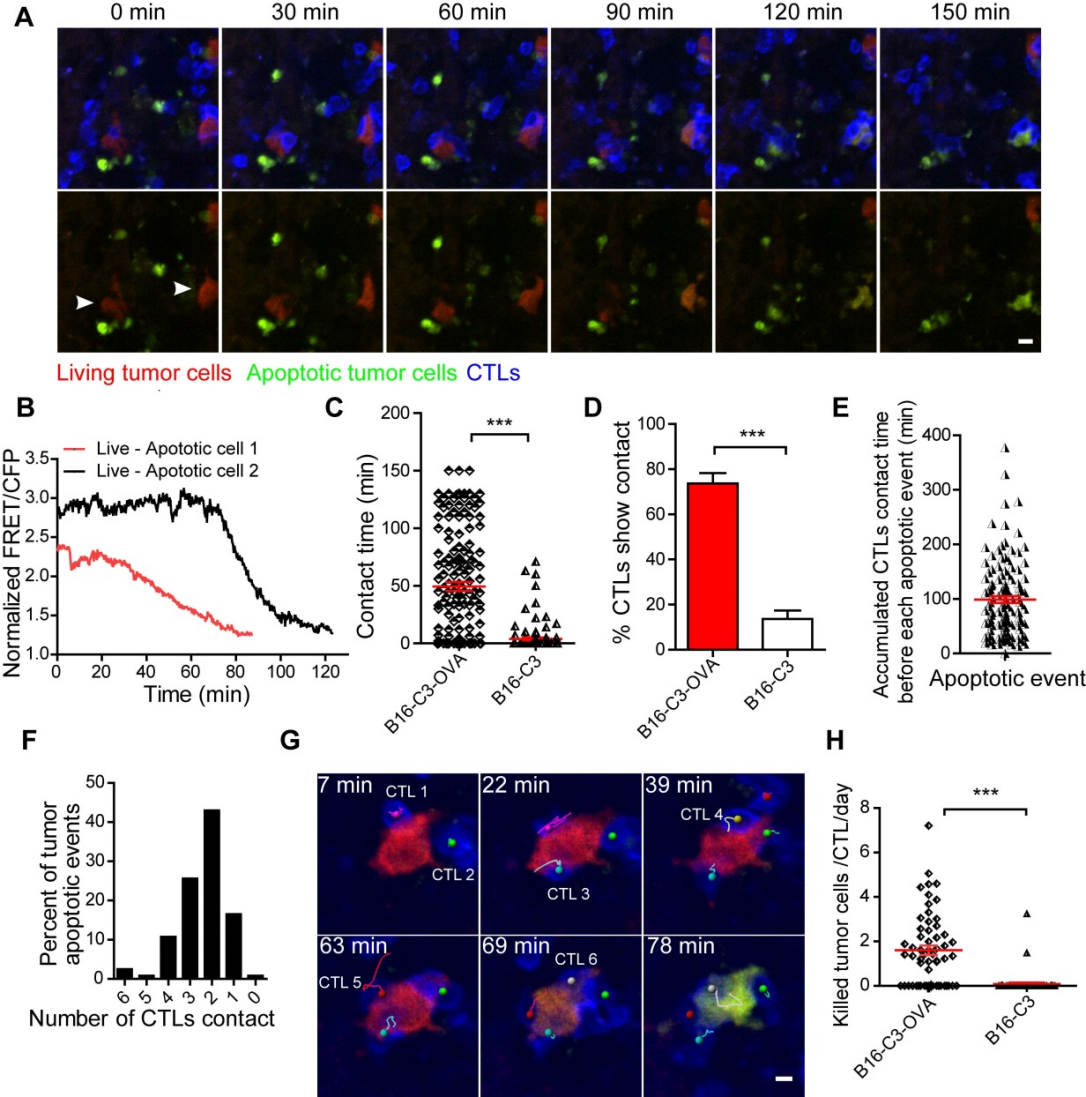
** CTLs show low killing efficiency of tumor cells in the liver.** (A) Intravital imaging reveals that prolonged contact between CTLs and tumor cells causes the loss of the FRET signal in tumor cells. Scale bar: 10 µm. (B) The loss of the FRET signal in individual tumor cells tracked over time. (C) The time of each CTL in contact with B16-C3 or B16-C3-OVA tumor cells. The unpaired t-test was used for statistical analysis, *** *P* < 0.001. (D) Percentages of CTLs that were in contact with tumor cells (data from C). The unpaired t-test was used for statistical analysis, *** *P* < 0.001. (E) The accumulated time for the interaction between each apoptotic tumor cell and CTLs, which represents the amount of contact time required by CTLs to induce a tumor cell from living to apoptosis. (F) The number of CTLs contacts that required to induce tumor cell apoptosis. (G) Example of six CTLs (magenta, green, cyan, yellow, red, and gray tracks) that were in contact with one tumor cell and then induced its apoptosis. Scale bar: 5 µm. (H) The efficiency of OT-I CTLs killing of tumor cells in the liver. A dot represents a video imaging for about 2 h. Data were pooled from 5-7 mice in each group from three independent experiments. The unpaired *t*-test was used for statistical analysis, *** *P* < 0.001.

Having demonstrated the sufficient infiltration of CTLs and their stable contact with tumor cells, we next sought to quantitatively evaluate the tumor killing capacity of CTLs *in vivo*. We extended the intravital imaging time to about 2 h for each video so that the whole process of CTL killing tumor cells in the liver could be observed. Apparently, living cells maintained a high FRET signal, and apoptotic cells displayed a sustained low FRET signal (Figure S3). The intravital FRET imaging data showed clearly that several CTLs attracted one tumor cell for more than one hour, and then caused the apoptosis of tumor cells with the loss of FRET signal (Figure 4A and Video 4). There were individual differences in the process of caspase-3 activity in tumor cells; the ratio of the fluorescent signal in the FRET channel to the CFP channel was decreased from a maximum to a minimum value around 20 minutes in most of the tumor cells, and in some tumor cells it lasted for 1 h (Figure 4B). The contact time for each CTL to tumor cell interaction was 49.4 ± 3.7 min in the B16-C3-OVA group, which was considerably longer than the 3.9 ± 1.1 min in the B16-C3 group (Figure 4C). Moreover, the number of CTLs, in percentage, that were in stable contact with tumor cells in the B13-C3-OVA group was significantly higher than that in the B16-C3 group (73.75% *vs* 13.6%, Figure 4D). These data suggest that the accumulated contact time of CTLs with tumor cells should be the key element in eliciting the apoptosis of tumor cells.

According to long-term intravital imaging data, we found that the accumulated CTL contact time for each apoptotic tumor cell was 98.9 ± 6.3 minutes (Figure 4E). 82.6% of tumor cells from living to apoptosis were contacted by several CTLs, in which 68.6% of tumor cells attacked by 2-3 CTLs and 14.0% of tumor cells attacked by 4-6 CTLs (Figure 4F). A typical example is shown in Figure 4G and Video 5, where 6 CTLs are in contact with one tumor cell before its death, indicating that it is a process in which multiple CTLs cooperate in fighting tumor cells.

To assess the capacity of CTLs to kill tumor cells quantitatively, we used a formula (N_death_ / N_CTLs_) × 24 / t_imaging_ (N_death_, number of tumor cells that from living to apoptosis in entire imaging video; N_CTLs_, mean CTL number for the whole time; t_imaging_, duration of the imaging video in hour) to calculate the number of tumor cells killed by each CTL per day. The quantitative data showed that each CTL killing liver metastatic tumor cells is 1.60 ± 0.21 tumor cells per day (Figure 4H). Compared with the CAR-T cell killing ability of lymphoma cells *in vivo* 30, CTLs were less efficient in killing solid tumor cells in the liver.

### The efficiency of CTLs killing tumor cells in the liver is less than in the spleen

Having demonstrated that CTLs possessed a low killing efficiency in the liver, possibly because of the immune-tolerant environment of the liver, we speculated that CTLs kill tumor cells in an organ with common immunity, such as the spleen, the classic immune organ, more efficient than in the liver. To compare CTLs killing efficiency to tumor cells in the liver and spleen, B16-C3-OVA cells (5 × 10^5^) were injected into the superficial zones of the liver and spleen, followed by intravenously transferred activated OT-I CTLs (2.5 × 10^6^) 3 days after tumor cell injection. Intravital imaging of the liver and spleen was performed 1 day after the adoptive transfer of CTLs. The trajectory, speed, and arrest coefficient of CTLs were similar in the liver and spleen (Figure S4A-C). The confinement ratio of CTLs in the liver was lower than that in the spleen (Figure S4D), and the mean displacement of CTLs in the liver in 10 min was shorter than that in the spleen (Figure S4E). These data indicate that the movement behaviors (speed and arrest coefficient) of CTLs in the tumor lesions of the liver and spleen were similar when tumor cells expressed homologous antigens. The difference in confinement ratio and mean displacement might influence the number of CTLs in the tumor microenvironment and the chance of tumor cells being exposed to CTLs.

**Figure 5:**
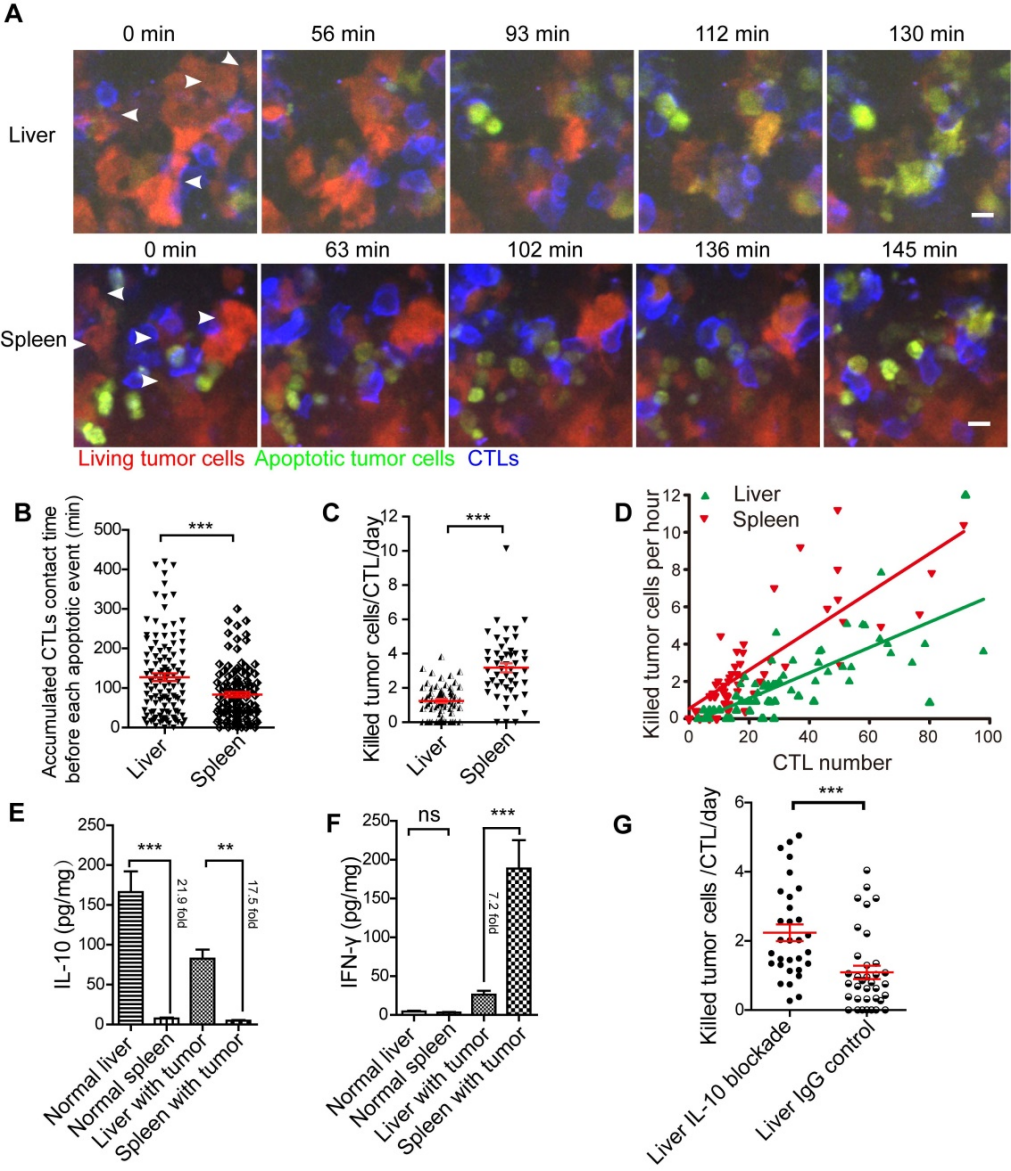
** The efficiency of CTLs killing of tumor cells in the liver is inferior to that in the spleen.** (A) Intravital imaging reveals the dynamic process of CTLs killing tumor cells in the liver and spleen. Scale bar: 10 µm. (B) The accumulated time for the interaction between each apoptotic tumor cell and CTLs in the liver and spleen, which represents the contact time required by CTLs to induce a tumor cell from living to apoptosis. The unpaired t-test was used for statistical analysis, *** *P* < 0.001. (C) The efficiency of CTLs killing of tumor cells in the liver and spleen. A dot represents a video imaging for about 2 h. Data were pooled from 7-9 mice in each group from three independent experiments and were expressed as mean ± SEM. The unpaired t-test was used for statistical analysis, *** *P* < 0.001. (D) The correlation between the number of tumor cell deaths per hour and the number of CTLs infiltration in the imaging area. A dot represents a video imaging for about 2 h. Pearson's correlation analysis was used. (E)The concentration of IL-10 in the liver and spleen. The unpaired t-test was used for statistical analysis, ** *P* < 0.01; *** *P* < 0.001, n=5. (F) The concentration of IFN-γin the liver and spleen. The unpaired t-test was used for statistical analysis, ns no-significant; *** *P* < 0.001, n=5. (G) The capacity of CTLs' killing tumor cells in the liver with IL-10 blockade. A dot represents a video imaging for about 2 h. Data were pooled from 5 mice in each group from three independent experiments and were expressed as mean ± SEM. The unpaired t-test was used for statistical analysis, *** *P* < 0.001.

We performed the intravital imaging of the liver and spleen to observe the entire process of tumor cells from living to apoptosis (Figure 5A, Video 6-7). We found that the accumulated CTL contact time for each apoptotic tumor cell in the liver was longer than that in the spleen (123.3 ± 11.7 min *vs* 83.0 ± 7.1 min, Figure 5B), indicating that compared with the spleen, CTLs took longer to kill tumor cells in the liver. We quantified the capability of CTL to kill tumor cells in the liver and spleen, and the data indicated that each CTL killed 1.24 ± 0.11 tumor cells per day in the liver, which was less efficient than that in the spleen (3.18 ± 0.26 tumor cells/CTL/day) (Figure 5C). The correlation analysis between CTLs number and death of tumor cells per hour showed that fewer tumor cells were killed in the liver than in the spleen, when both tumor microenvironments contained the same number of CTLs (Figure 5D), possibly because the immune-tolerant environment of the liver suppresses CTL killing efficiency.

Next, we analyzed the expression of cytokines in the liver and spleen of mice inoculated with tumor cells one day after the transfer of CTLs. Our findings showed that IL-10 concentration in the liver was significantly higher than that in the spleen, with a 21.9-fold in normal mice and 17.5-fold in tumor-bearing mice (Figure 5E). There was no difference in IFN-γ concentration between the liver and spleen in normal mice. Expectedly, after CTL transfer, the concentration of IFN-γ in the spleen of tumor-bearing mice was significantly higher (7.2-fold) than that in the liver (Figure 5F). IL-6 and TNF-α concentrations in the liver were considerably higher than those in the spleen of normal mice, but not markedly different in the liver and spleen with tumors (Figure S4F-G). IL-10 is an immunosuppressive factor playing an important role in the immune-tolerant status of the liver. To further confirm that the immune-tolerant status is the reason to suppress CTL killing in the liver, we blocked the function of IL-10 in the liver by intraperitoneally injected an IL-10 neutralizing antibody. The quantitative data of intravital imaging revealed that the capacity of CTLs' killing tumor cells in the liver was significantly increased from 1.09 ± 0.10 tumor cells/CTL/day to 2.24 ± 0.24 tumor cells/CTL/day after IL-10 blockade (Figure 5G). The CTLs' killing capacity in the liver with IL-10 blockade was lower than that in the spleen (3.18 ± 0.26 tumor cells/CTL/day), implying that IL-10 is one of the reasons to suppress CTL killing. These results suggest that CTLs ability to kill tumors is restrained in the liver and can be reversed by blocking immunosuppressive cytokine.

### *In vivo* imaging of calcium signaling reveals early response of CTLs killing tumor cells

The activation of caspase-3 is at the effect stage in tumor cell apoptosis. We also wanted to reveal the trigger time of tumor cells to respond to CTLs *in vivo*. We know that the action of perforin leads to calcium influx in target cells when CTLs kill tumor cells 6, and this influx occurs earlier than the activation of caspase-3. To establish how long tumor cells took to respond to the contact of CTLs in the early stage, we established a model for the intravital imaging of calcium signaling in tumor cells that co-expressed the Twitch2B 31 calcium probe and OVA in B16 cells, and another control cell line that only expressed the Twitch2B probe. Confocal imaging confirmed that calcium influx in tumor cells was characterized well by the Twitch2B probe, with orange indicating cells with low calcium and red indicating cells with high calcium (Figure S5A-B).

**Figure 6:**
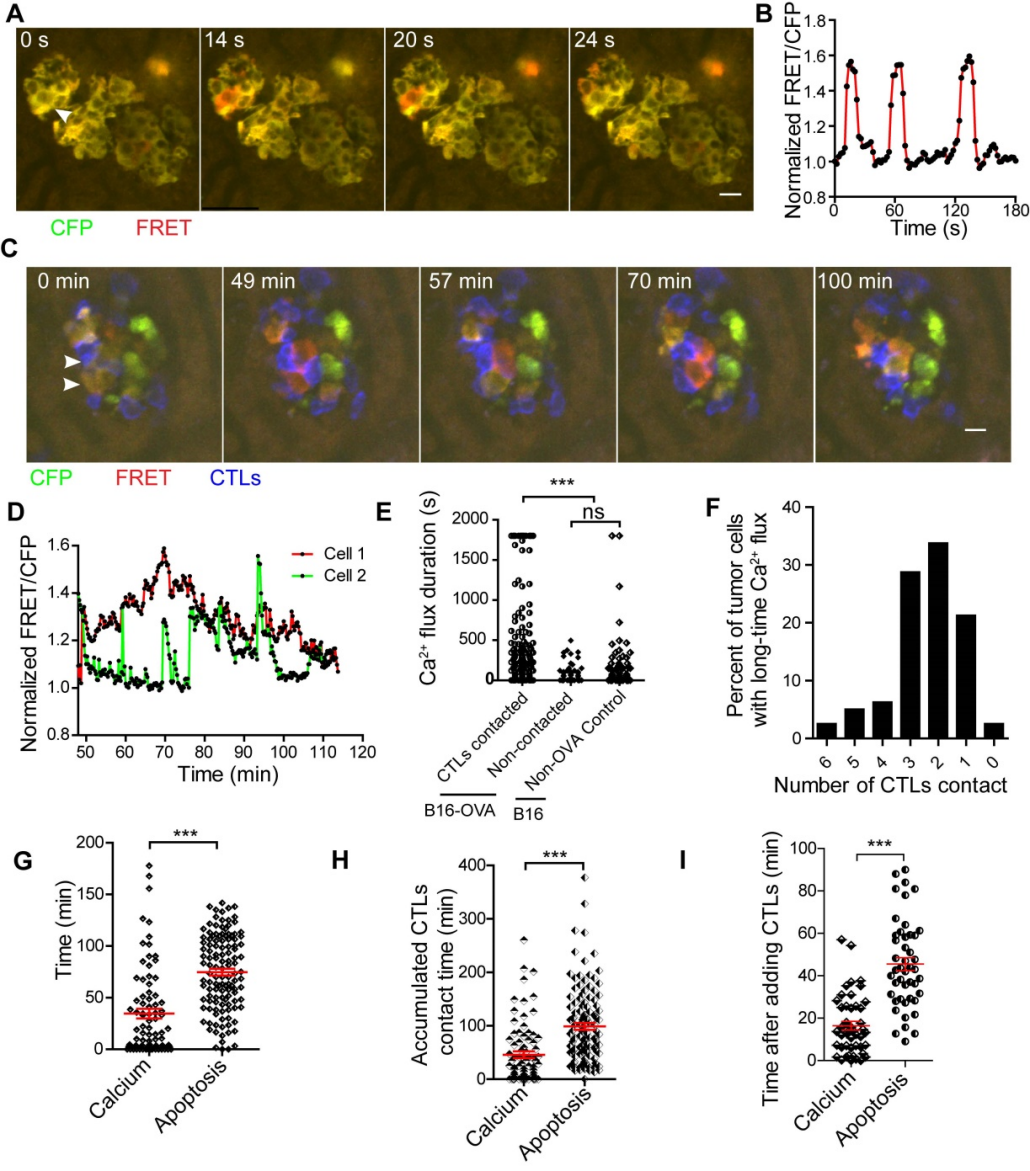
** CTLs induce prolonged calcium influx in the process of killing tumor cells in the liver.** (A) Tumor cells show short spontaneous calcium influx. B16 cells are co-expressing with the FRET calcium sensor Twitch2B and ovalbumin. (B) Calcium influx of tumor cells, indicated by the arrow in A. (C) Intravital imaging of how CTLs contact elicits prolonged calcium influx in tumor cells. (D) Prolonged calcium flux when tumor cells are killed by CTLs, indicated by the arrow in C. (E) The accumulated calcium influx time in each tumor cell with or without contact with CTL in a 30-min imaging video. Data were pooled from 4-6 mice in each group from three independent experiments and were expressed as mean ± SEM. The unpaired t-test was used for statistical analysis, ns no-significant; *** *P* < 0.001. (F) The number of CTLs that were in contact with tumor cells before the occurrence of prolonged calcium influx in tumor cells. (G) The intravital imaging time before the tumor apoptosis signaling change or prolonged calcium signaling occurrence. The unpaired t-test was used for statistical analysis, *** *P* < 0.001. (H) The accumulated contact time between CTLs and tumor cells before the tumor apoptosis signaling change or prolonged calcium signaling occurrence* in vivo*. Data were pooled from 6-7 mice in each group from three independent experiments and were expressed as mean ± SEM. The unpaired t-test was used for statistical analysis, *** *P* < 0.001. (I) The time the tumor apoptotic signaling change or prolonged calcium signaling occurrence after adding CTLs *in vitro*. The unpaired t-test was used for statistical analysis, n = 46, *** *P* < 0.001.

As shown by the schedule in Figure 2A, we prepared a liver metastasis model and performed intravital imaging to monitor calcium signaling changes *in vivo*. We found that tumor cells had spontaneous short-time calcium influxes even without the adoptive transfer of CTLs (Figure 6A-B, and S5C, Video 8), and the duration of each calcium influx was less than 30 s. Tumor cells in contact with CTLs may appear a prolonged calcium influx, with calcium signaling lasting longer than 30 s (Figure 6C-D, and Video 9).

We analyzed the accumulated time of calcium signaling generated in each tumor cell during 30-min of imaging. The accumulated time of calcium influx in tumor cells that were in contact with CTLs was 446.1 ± 44.0 s (Figure 6E), which was significantly longer than that of tumor cells that had no contact with CTL in the B16-OVA + CTLs group (35.4 ± 7.2 s), and that of tumor cells in the B16 + CTLs control group (52.4 ± 12.5 s). When B16-OVA tumor cells were exposed to CTLs, 41% of the tumor cells (data from Figure 6E) appeared to have prolonged calcium influx, indicating that the efficiency of CTLs in killing tumor cells *in vivo* is heterogeneous.

Through long-term imaging (about 2 h) of the tumor microenvironment in the liver, we found that 76.3% of the tumor cells with prolonged calcium influx were in contact with more than one CTL (Figure 6F). Prolonged calcium influx in the tumor cells exposed to CTLs occurred about 40 min before the occurrence of apoptosis (mean 34.6 ± 4.7 min *vs* 74.8 ± 3.3 min, Figure 6G). The accumulated CTL contact time to elicit prolonged calcium influx in B16-OVA cells was 45.9 ± 6.3 min, which was shorter than the accumulated CTL contact time required to induce apoptosis (98.9 ± 6.3 min, Figure 6H). We also confirmed the time difference between prolonged calcium influx and caspase-3 activity during CTL-mediated tumor cell killing *in vitro*. R-CaMP1.07 calcium probe 40, a genetically encoded red fluorescent Ca^2+^ indicator, was transfected in B16-C3-OVA cells. The absorbance and emission peaks of R-CaMP1.07 were 562 nm and 584 nm, respectively; hence, tumor cells could be simultaneously stained with the C3 apoptosis probe. The occurrence of CTL-induced prolonged calcium influx in tumor cells (16.5 ± 2.0 min) also earlier than the caspase-3 activity (45.5 ± 3.0 min)* in vitro* (Figure 6I, Figure S6A-B). The time difference between prolonged calcium signaling and apoptosis signaling was 29 ± 2.4 min (Figure S6C). These data imply that CTLs elicited prolonged calcium influx in tumor cells as a prerequisite step for tumor cell apoptosis.

## Discussion

As the primary effector cells for killing tumor cells, CTLs play a key role in eliminating tumors. However, the efficiency and dynamics of CTLs killing of tumor cells* in vivo*, especially in the liver, an organ with a high incidence of tumorigenesis and metastasis due to its regional immunity, is not clear. Here, we visualized how CTLs kill tumor cells in the liver by intravital molecular imaging. We proposed a series of quantitative parameters to evaluate the capacity of CTLs to kill tumor cells accurately by using the FRET imaging of caspase-3 activity and calcium signaling *in vivo*. Our findings confirmed that CTL killing of tumor cells *in vivo* is a low-efficiency process and requires multiple CTLs in synergistic action. The killing ability of CTLs is also associated with the immune status of the corresponding organ. CTLs trigger a prolonged calcium influx (> 30 s) in tumor cells, which occurs earlier around 40 min before apoptosis.

CTLs secrete perforin to induce calcium influx in tumor cells and calcium signaling is also involved in other stages of apoptosis 41. In our results, prolonged calcium influx in tumor cells occurred earlier than caspase-3 activity when CTLs killed tumor cells (Figure 6G). Our method evaluated the ability of CTLs to kill tumors *in vivo*, where the caspase-3 activity is the effect stage and the calcium influx is the early response stage. We analyzed the ability of CTLs to kill tumor cells *in vivo* using a series of parameters, including the accumulated contact time of CTLs with tumor cells, the number of tumor cells killed by CTLs every day, tumor cells early-stage response time to CTL (calcium signaling), and the effector stage time of tumor apoptosis (caspase-3 activity signaling). Therefore, we established a multi-parameter system for the* in vivo* evaluation of the capacity of CTL-induced tumor cell apoptosis.

Different anatomical sites affect the ability of CTLs to kill target cells 30, 42. Our results indicated that the capacity of CTLs to kill tumor cells was significantly different between the liver and the spleen, possibly due to the immune microenvironments of the liver and spleen. As an immune-tolerant organ, the immune microenvironment of liver is more conducive to the establishment of the tumor microenvironment, and promote CTL dysfunction and inhibit CTL activity 43, 44. The liver region has high concentrations of immunosuppressive cytokines, such as IL-10, which inhibit CTLs to secrete IFN-γ that may affect the ability of CTLs to kill tumor cells. In addition, different immune cell types in the liver and spleen may also affect the immune microenvironment. For instance, the Kupffer cells and liver sinusoidal endothelial cells (LESC) in the liver help maintain the liver's immune-tolerant microenvironment 44, which may inhibit the capacity of CTLs to kill tumor cells. The spleen is a critical immune organ responsible for T cell maturation and function. The spleen contains a large number of DCs, macrophages, and Th cells 45, which facilitate CTL activation and function. Our findings showed that CTLs killed 1.60 ± 0.21 tumor cells per day in the liver, which is lowly efficient, compared with CTL ability to kill virus-infected cells 46. It is due to that tumor cells have mechanisms to resist CTL killing 47. CTL killing efficiency of melanoma tumor cells *in vivo* is also lower than CART efficiency in killing lymphoma cells* in vivo* 30. This difference in outcome may stem from the dissimilarity in the signaling pathways of CTL and CART, the infiltration efficiency of CTLs in solid tumors, and the immunosuppressive microenvironment of solid tumors.

ACT therapy for liver tumors is a promising prospect. In addition to the general obstacles ACT therapy face in the treatment of solids tumor, such as antigen heterogeneity and tumor microenvironment resistance 48, ACT therapy for liver tumors also faces challenges with the liver's immune-tolerant microenvironment. Our results demonstrate that CTL has a good therapeutic effect in OVA-expressing tumors, significantly lengthening the survival time of mice with liver metastasis (Figure 2B). This healing effect is probably due to the CTLs ability to reach liver tumor lesions and kill tumor cells effectively. Our results suggest that CTLs are limited in their capacity to kill tumor cells in the liver. If the immune microenvironment of the liver changes from immunosuppression to activation, it will boost the therapeutic effect of ACT in liver tumors.

In summary, our study shows the dynamic process of the clearance of tumor cells by CTLs in the liver, quantifies the capacity of CTLs killing tumor cells, reveals the synergy of CTLs in killing tumors, and highlights the impact of organ microenvironment on CTLs function. This research establishes the characteristics of CTLs killing tumor cells *in vivo*, and these characteristics suggest a superior CTL density and a more potent CTL killing ability is required to achieve the desired therapeutic effect in tumor immunotherapy.

## Materials and Methods

### Mice

C57BL/6 mice were obtained from Hunan Slack King of Laboratory Animal Co., Ltd (Changsha, China). OT-I mice and mT/mG mice were purchased from the Jackson Lab (Bar Harbor, USA). To generate OT-I × mT/mG mice, OT-I mice were hybridized with mT/mG mice in which cell membrane-localized tdTomato fluorescence expression is widespread in cells/tissues. All mice were bred at the animal facility of WNLO-HUST under specific-pathogen-free (SPF) conditions and used in 7-10 weeks old. All animal studies were approved by the Animal Experimentation Ethics Committee of Huazhong University of Science and Technology (IACUC Number: 843).

### Tumor cells

The B16F10 melanoma cell line was purchased from the BOSTER Company (Wuhan, China). The Twitch2B calcium sensor was a gift from Professor Oliver Griesbeck (Martinsried, Germany). The ovalbumin gene was a gift from Sandra Diebold & Martin Zenke (Addgene plasmid #64599). The caspase-3 sensor, calcium sensor and ovalbumin were introduced into B16 cells using a PiggyBac transposon system (a gift from Dr. Xiaohui Wu, Fudan University, Shanghai, China). All cell lines were negative for mycoplasma, as detected by the MycoProbe Mycoplasma Detection Kit (R&D Systems, USA). All cell lines mentioned above were cultured in RPMI 1640 containing 10% FBS (Gibco, USA) and under 5% CO_2_ at 37 °C in a constant temperature incubator (Thermo, USA).

### Plasmid construction

To construct PiggyBac-C3-IRES-OVA, we synthesized primers containing the DEVD linker. Primer sequences were as follows: Forward primer 5′-GCATGCATGACCAACTGACAGAAGAGGATGAGGTCGATGGTGGTGAGCTC-3′ and reverse primer 5′-GAGCTCACCACCATCGACCTCATCCTCTTCTGTCAGTTGGTCATGCATGC -3′. The forward and reverse primers were mixed in equal proportions, heated at 94 °C, and cooled at 10 °C. The DEVD linker was used to link mCerulean3 and cpVenus in the pcDNA3.1(+) vector. Next, the IRES and OVA sequences were constructed and cloned into the pcDNA3.1(+)-C3 vector. Finally, the C3-IRES-OVA sequence was digested with Afl II and Pme I, and constructively cloned into the PiggyBac vector.

### RNA extraction and semi-quantitative PCR

Total RNA was extracted from tumor cells using a TRIzol reagent (Invitrogen, USA). Equal amounts of RNA were reverse transcribed into cDNA using the PrimeScript RT Reagent Kit with gDNA Eraser (Takara, Japan). Primer sequences were as follows: Ovalbumin forward primer 5′-TGGCACATCTGTAAACGTTC-3′ and reverse primer 5′-CACTCTGAAAGGCATTGCTTG-3′; β-actin forward prime 5′-GTGACGTTGACATCCGTAAAGA-3′ and reverse primer 5′- GCCGGACTCATCGTACTCC-3′. PCR annealing temperature was 56 °C, and PCR products were visualized employing electrophoresis on a 1.5% (w/v) agarose gel containing 0.01% GelRed (Biosharp, China) and imaged with a gel imaging system (Bio-Rad, USA).

### The adoptive transfer of activated OT-I CD8^+^ T cells

OT-I × mT/mG mice (7-10 weeks) were sacrificed by cervical dislocation and disinfected, and the lymph node cells were cultured in RPMI 1640 medium, with 10% FBS, 10 ng/ml OVA_257-264_ peptide and 100 U/ml IL-2 (PeproTech) for 3 days. Next, the cells were harvested and washed 3 times with RPMI 1640 medium, and cultured further in RPMI 1640 medium containing 10% FBS and 100 U/ml IL-2 for another 3 days. The cells were then harvested again and purified using a Mouse CD8 T cell Enrichment Kit (Thermo, USA). Recipient mice received an adoptive transfer of 2.5×10^6^ activated OT-I CD8^+^ T cells in 150 μL of PBS by intravenous injection.

### Animal model

For the liver metastasis model, tumor cells were harvested at the exponential phase, and 5 × 10^5^ tumor cells were resuspended in 100 μL of PBS. Mice were anesthetized with a mixture of 10 mg/kg xylazine and 100 mg/kg ketamine hydrochloride (Sigma, USA), and dehaired with hair clippers and depilatory cream. The spleen of the mouse was tied and divided into two halves, and 100 μL of tumor cells were inoculated into the half of the spleen. Ten minutes later, the half of the spleen that was injected with tumor cells was resected. Three days later 2.5×10^6^ activated OT-I CTLs were adoptively transferred into liver metastasis mice. On the fourth day after tumor cells inoculation, the livers of the mice were collected for cryosection, and another batch of mouse liver was collected on day nine for H&E.

For the liver and spleen *in situ* models, 5 × 10^5^ tumor cells were resuspended in 100 μL of PBS. Mice were anesthetized and dehaired. The spleen or the liver was exposed by surgery, and 100 μL of tumor cells were inoculated into the superficial zones of the spleen and liver. Three days later, 2.5 × 10^6^ activated OT-I CTLs were adoptive to tumor-bearing mice. The mice were used for intravital imaging on the fourth day after tumor cell inoculation.

### Confocal imaging

Cells were cultured in 28 mm glass-bottomed dishes (NEST, China) and imaged with an LSM710 confocal microscope with a dry 20×/0.8 NA objective (Zeiss, Germany). The fluorescent signal of mCerulean3 (CFP) and FRET were detected using excitation at 458 nm and the fluorescent signal of cpVenus (YFP) was imaged using excitation at 514 nm. Emissions were recorded at 460-505 nm (CFP) and 515-563 nm (FRET and YFP).

To measure the emission spectrum of the C3 probe in normal and apoptotic cells, B16-C3 cells were cultured in 28 mm glass-bottomed dishes, and apoptosis inducers (Apopisa and Apobid, 1: 1000; Beyotime) were used to induce cell apoptosis. We performed confocal spectral scanning imaging 24 h after adding apoptosis inducers. The C3 emission spectra were recorded between 461 and 629 nm with excitation at 458 nm. The dynamic range was calculated based on the maximum emission peaks of CFP and FRET. The dynamic range of the mCerulean3 and cpVenus fluorescent protein pair in the cell was calculated as follows 49: (R_live cell_-R_apoptosis cell_)/R_apoptosis cell_, R = the emission peak intensity of FRET/the emission peak intensity of CFP.

Livers with tumor metastasis were fixed in 4% paraformaldehyde, dehydrated in 30% source, frozen in OCT compound, and then sectioned (20 μm) on a CM1950 cryostat (Leica, Germany). Liver sections were imaged on an UltraVIEW VoX Spinning Disk inverted Confocal Microscope (PerkinElmer, USA). The fluorescent signals of CFP and FRET were imaged using excitation at 440 nm, and the emissions were recorded at 450-500 nm (CFP) and 510-560 nm (FRET). The fluorescent signal of tdTomato (OT-I CTLs) was imaged using excitation at 561 nm and recorded at 580-650 nm. Data were collected with velocity software and analyzed further with the Fiji software.

To simultaneously assess the timing of calcium signaling and apoptosis signaling in the same cell *in vitro*, B16-C3-OVA cells was transfected with the pN1-R-CaMP1.07 plasmid using Lipofectamine 2000 (Invitrogen) according to the manufacturer's manual. After adding activated tdTomato-expressing OT-I CTLs, tumor cells were imaged using an UltraVIEW VoX Spinning Disk inverted Confocal Microscope (PerkinElmer, USA). The fluorescent signals of CFP and FRET were imaged using excitation at 440 nm, and the emissions were recorded at 450-500 nm (CFP) and 510-560 nm (FRET). The fluorescent signals of tdTomato (OT-I CTLs) and R-CaMP1.07 were imaged using excitation at 561 nm and recorded at 580-650 nm. Data were collected with velocity software and analyzed further with the Fiji software.

### Intravital imaging

Intravital imaging was performed using an UltraVIEW VoX Spinning Disk inverted Confocal Microscope (PerkinElmer, USA) with a dry 20×/0.75 NA objective (Olympus, Japan) 1 day after activated CTLs were transferred into tumor-bearing mice. The mice were first anesthetized with a mixture of 10 mg/kg xylazine and 100 mg/kg ketamine hydrochloride. Next, the liver or the spleen was exposed by surgery, and the mouse was placed in a box maintained at a constant temperature of 37℃. The liver or the spleen of the mouse was attached to a slide and moistened with pre-warmed PBS. Mice were then anesthetized with 0.5-1.0% isoflurane in oxygen flow at 0.6 L/min controlled by a small animal anesthesia machine (RWD, China). The fluorescent signals of CFP and FRET were imaged using excitation at 440 nm, and the emissions were recorded at 450-500 nm (CFP) and 510-560 nm (FRET). The fluorescent signal of tdTomato (OT-I CTLs) was imaged using excitation at 561 nm, and its emission was recorded at 580-650 nm. Data were collected with velocity software and videos were processed further with Imaris (version 7.6.1, Bitplane) and the Fiji software. Trajectories of individual OT-I CTLs were plotted following the alignment of their starting positions by MATLAB 50.

### Calculating CTLs killing efficiency

To calculate CTL killing efficiency, we used Imaris spot detection to determine the number of CTLs per imaging volume for the entire time. We manually counted the number of dead tumor cells that FRET changed from living to apoptosis. The number of tumor cells killed by each CTL in 24 hours was calculated on average as follows 46: (N_death_ / N_CTLs_) × 24 / t_imaging_ (N_death_, number of tumor cell that from living to apoptosis in the entire imaging video; N_CTLs_, mean CTL number for the entire time; t_imaging_, duration of the imaging video in hours).

To determine CTL contact time with tumor cells, we calculated the contact time 51 of each CTL with tumor cells throughout the imaging process. We defined a 0 min contact time as no contact between CTL and tumor cells.

To determine the accumulated CTL contact time for each apoptotic tumor cell, we calculated the contact time of all CTLs that attacked one tumor cell to induce caspase-3 activity in about 2 h of imaging video.

To determine the accumulated CTL contact time for each prolonged calcium influx tumor cell, we calculated the contact time of all CTLs that attacked one tumor cell to induce the first prolonged calcium influx (> 30s) in about 2 h of imaging video.

### Cytokine detection

The livers and spleens of tumor-bearing mice were collected, and their masses were measured. Then, tissue samples were lysed in NP-40 lysis buffer (5 μL/mg tissue, BOSTER, China) and freshly supplemented with a protease inhibitor cocktail (Beyotime, China). Lysates were aliquoted and stored at -80°C until analysis. Samples were assayed using the LEGENDplex Mouse Th1 Panel (BioLegend) according to the instructions of the accompanying manual. Data were analyzed with the Legendplex software (BioLegend).

### IL-10 neutralization *in vivo*

Neutralization of IL-10 was performed using an anti-IL-10 antibody (JES5-2A5, BioXCell) or isotype control IgG antibody (HRPN, BioXCell). Mice were intraperitoneally injected with 200 μg antibody in 12 h and 3 h before intravital imaging, respectively. Intravital imaging was performed 1 day after transferring activated OT-I CD8^+^ T cells into mice (4 days after tumor inoculation).

### Statistical analysis

Experimental data are presented as means ± SEM. Linear charts, histograms, and correlation analyses were performed using GraphPad Prism (GraphPad Software, USA) or Origin 9 (OriginLab, USA). The two-way ANOVA analysis was used for flow cytometry analysis. The log-rank test was used for the survival curve analysis. For comparisons between two groups, the unpaired *t*-test was used. For correlation analysis, Pearson's correlation analysis was used. Differences between or among groups were denoted by ns no-significant, * *P* < 0.05, ** *P* < 0.01, *** *P* < 0.001.

## Supplementary Material

Supplementary figures and tables.

Supplementary videos.

## Abbreviations

TCR: T-cell receptor
TILs: tumor-infiltrating lymphocytes
OT-I: ovalbumin T-cell receptor I
OVA: ovalbumin
IRES: internal ribosome entry site
LSEC: liver sinusoidal endothelial cells
H&E: hematoxylin and eosin
FRET: fluorescence resonance energy transfer
ERK: extracellular signal-regulated kinase
DEVD: aspartic acid - glutamic acid - valine - aspartic acid
CTLs: cytotoxic T lymphocytes
CAR: chimeric antigen receptor
ACT: adoptive cell therapy
