# Supplementary material for: Intravital molecular imaging reveals the restrained capacity of CTLs in the killing of tumor cells in the liver
Source: Theranostics. 2021 Jan 1;11(1):194–208. doi: 10.7150/thno.44979 (PMC7681101; doi:10.7150/thno.44979)
Supplement: Supplementary file 1 — Supplementary figures and tables. [file thnov11p0194s1.pdf]

Supplemental material

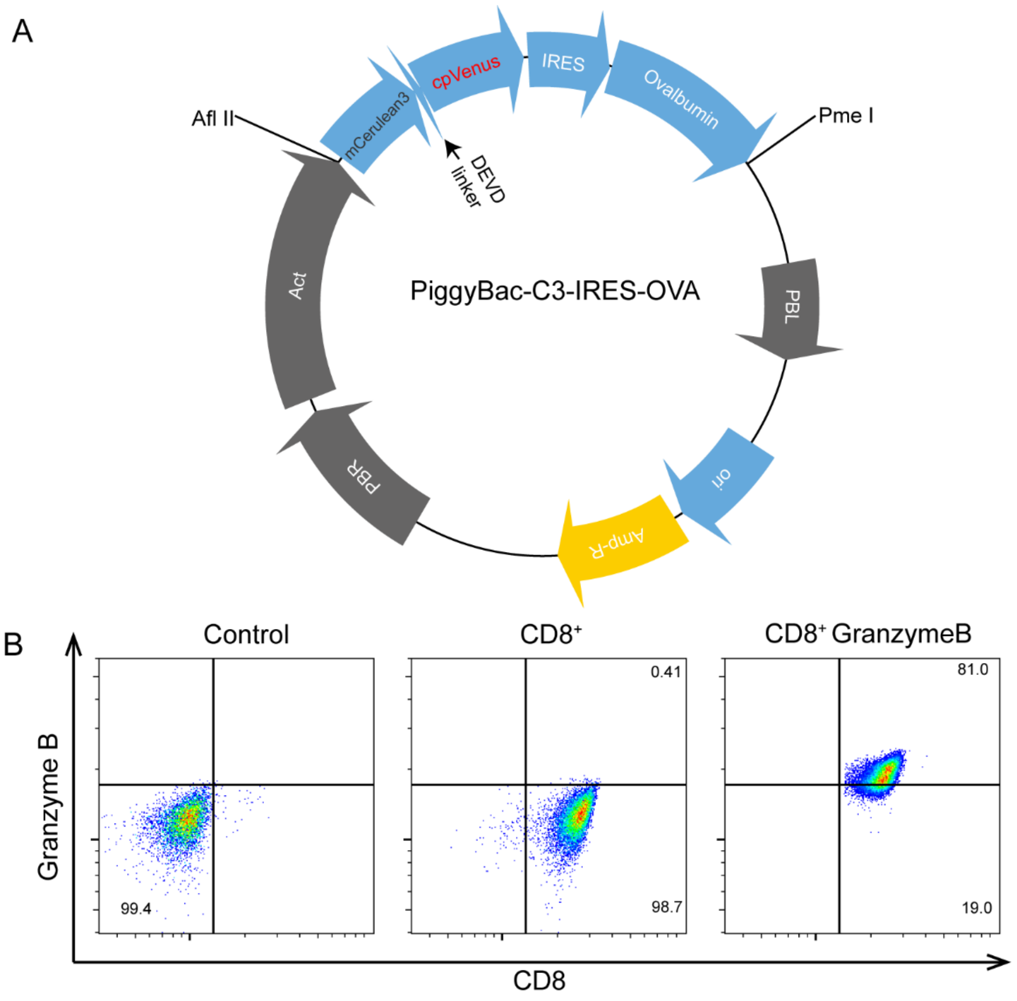

Figure S1. (A) The plasmid map of PiggyBac-C3-IRES-OVA. (B) Flow cytometry analysis for granzyme B expression in transferred $CD8^+$ T cells. After *in vitro* activation for 6 days, activated OT-I $CD8^+$ T cells were harvested and purified using a mouse CD8 T cell enrichment kit. Most the transferred OT-I $CD8^+$ T cells expressed granzyme B.

.

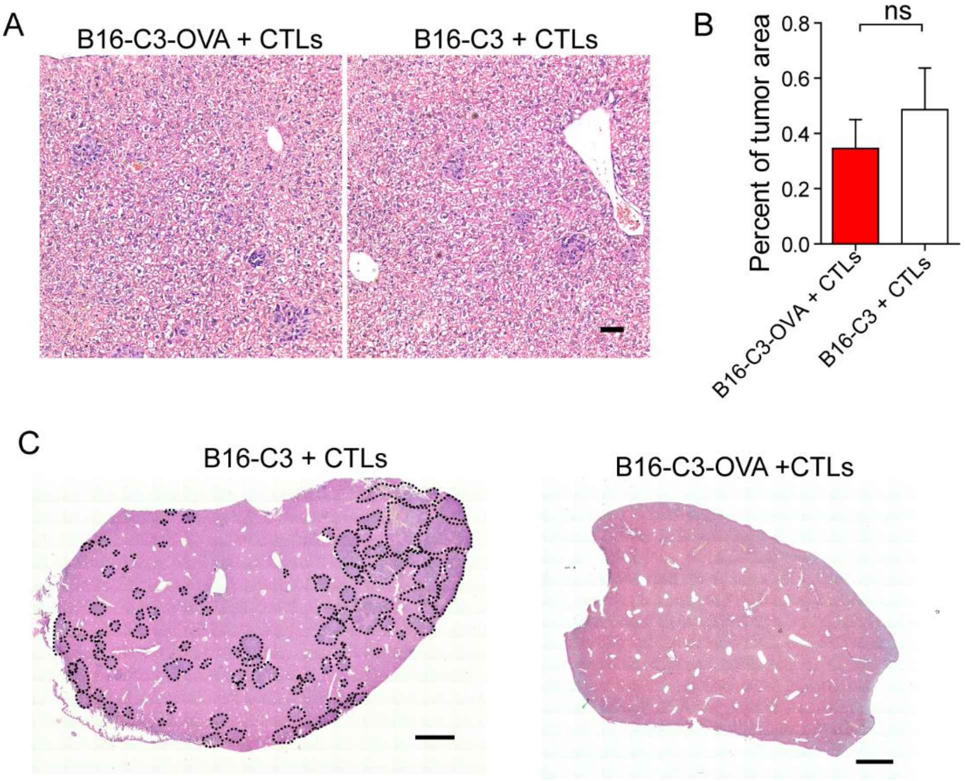

Figure S2. (A) Representative images of hematoxylin and eosin (H&E) staining of liver sections from liver metastasis mice on day 4; mice were transferred with activated OT-I CD8$^+$ T cells on day 3. Scale bar: 50 µm. (B) The percentages of the tumor metastasis areas in the liver lobe. The data in each group were pooled from 5 mice. The unpaired t-test was used for statistical analysis, ns no-significant. (C) Large images of hematoxylin and eosin (H&E) staining of liver lobe sections from the mice with B16-C3 and B16-C3-OVA liver metastases on day 9. Scale bar: 1000 µm.

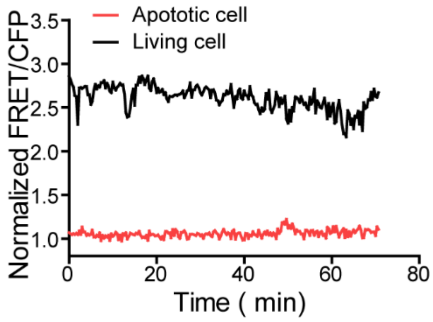

Figure S3. Normalized FRET/CFP ratio of living cell and apoptotic cell.

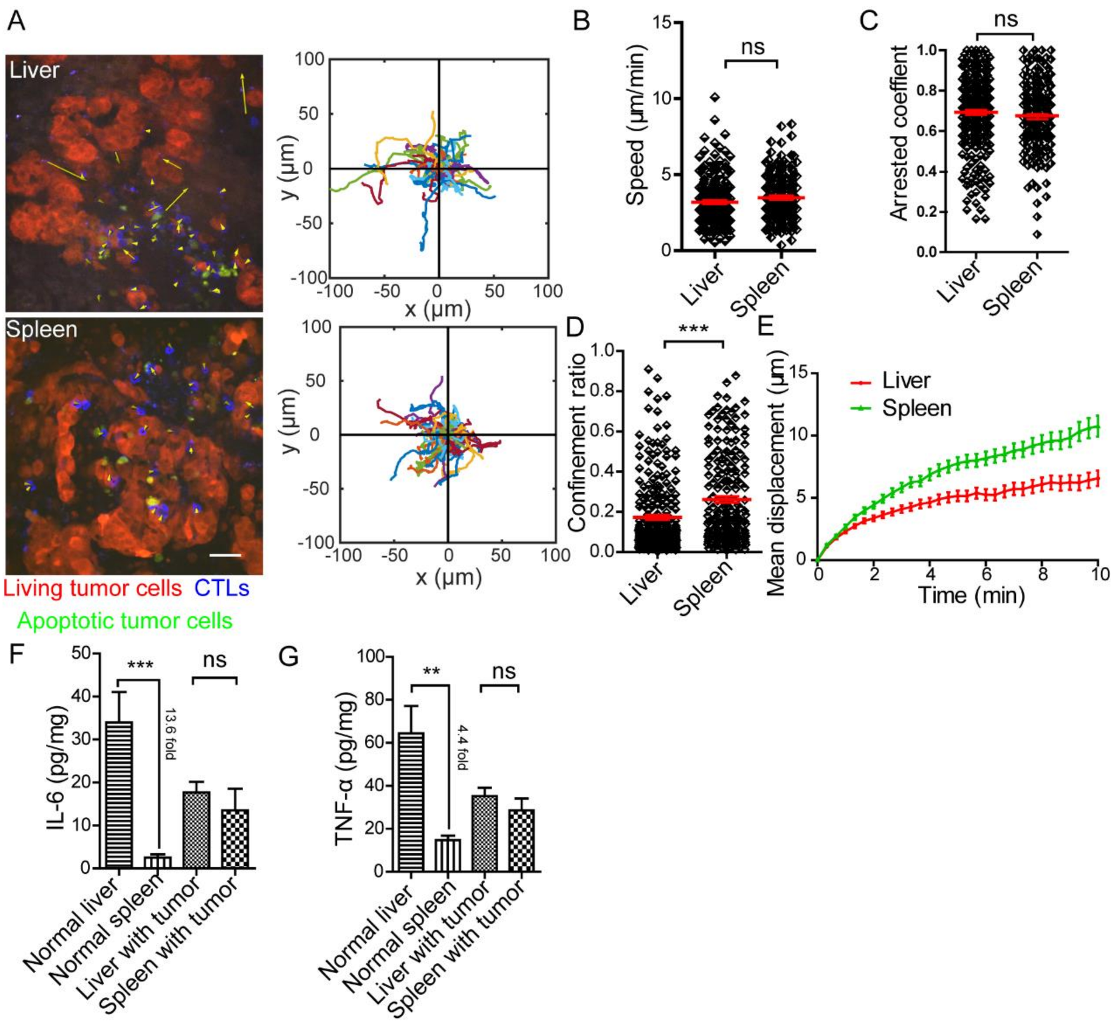

Figure S4. (A) A typical image of CTLs' displacement in the liver and spleen tumor areas. The scale bar is 40 μm, and arrows represent CTLs' displacement. The trajectories of CTLs' movements were tracked and extracted following the alignment of their starting positions. (B-D) Statistical analysis of B) the mean speed C) the arrest coefficient, D) the confinement ratio, each point represents a CTL from a 30-min video. Data were pooled from 7-9 mice in each group from three independent experiments and were expressed as mean ± SEM. The unpaired t-test was used for statistical analysis, *** $P < 0.001$. (E) The mean displacement of CTLs track for 10 mins. (F) The concentration of IL-6 in the liver and spleen. The unpaired t-test was used for statistical analysis, ns no-significant; *** $P < 0.001$, n=5. (G) The concentration of TNF-α in the liver and spleen, n = 5, The unpaired t-test was used for statistical analysis, ** $P<0.01$; ns, no significant.

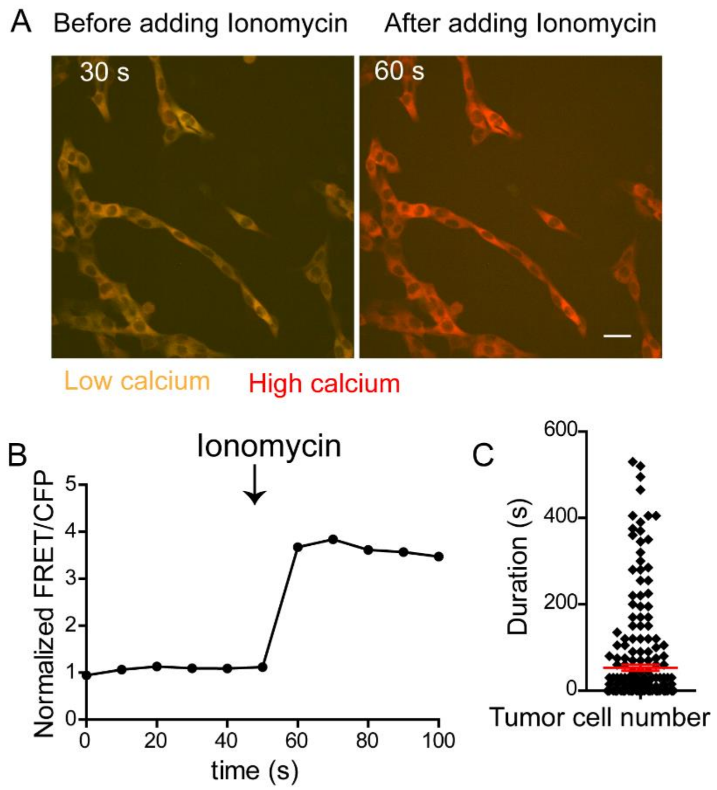

Figure S5. (A) The FRET imaging of the Twitch2B calcium probe-expressing tumor cells, that treated with ionomycin *in vitro*. (B) Ionomycin induces increasing calcium signaling (FRET/CFP) in tumor cells. (C) The accumulated time of spontaneous calcium influx in each tumor cell from a 30-min imaging video (dots represent cells, n = 239 tumor cells from four mice).

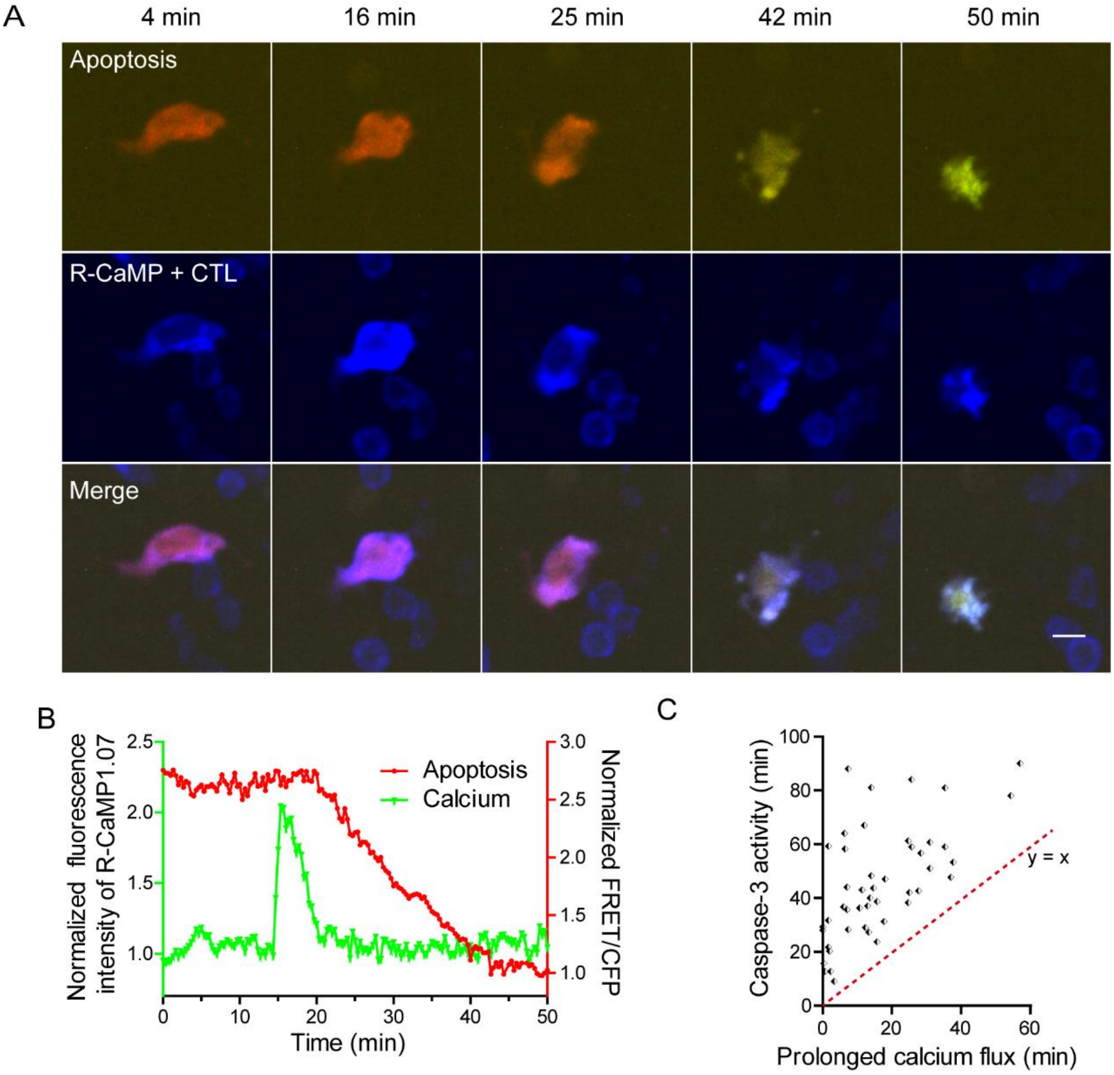

Figure S6. Dynamic visualization of the change of C3 probe FRET signaling and calcium signaling during activated CTL-mediated killing of B16-C3-OVA cells *in vitro*. (A) Dynamic visualization the change of the apoptotic signaling, calcium signaling by time lapse microscopy *in vitro*. In order to observe the apoptotic signaling and calcium signaling in one cell, the B16-C3-OVA cells were transfected with R-CaMP1.07 calcium probe. The CTLs/ B16-C3-OVA ratio is 5:1. Scale bar: 10 μm. (B) Apoptotic signaling, calcium signaling change curve with time during CTL-mediated tumor cells killing. Prolonged calcium influx occurs before caspase-3 activity and the apoptosis C3 probe FRET lost. (C) The time difference between prolonged calcium signaling and apoptotic signaling. Each point represents the time of the occurrence of prolonged calcium signaling and caspase-3 activity in a cell; n =46.

Video 1. Dynamic visualization of the FRET signal change when B16-C3-OVA cells are killed by activated CTLs *in vitro*. Activated OT-I CTLs were added to the dishes containing B16-C3-OVA cells, after which imaging was performed immediately. The CTLs/ B16-C3-OVA ratio is 20/1. Living tumor cells appear in red, apoptotic tumor cells appear in green, and CTLs appear in blue. Scale bar: 5 μm. Total duration = 20 min.

Video 2. The migration behavior of CTLs in the liver with B16-C3-OVA tumor metastases. 5×10^5 B16-C3-OVA cells were injected into the hemispleen of C57BL/6 mice, followed by the intravenously transferred 2.5×10^6 activated OT-I CTLs into mice three days later. Time-lapse intravital imaging of the liver metastasis microenvironment was taken one day after OT-I CTLs transfer. Living tumor cells appear in red, apoptotic tumor cells appear in green, and CTLs appear in blue. Scale bar: 40 μm. Total duration = 30 min.

Video 3. The migration behavior of CTLs in the liver with B16-C3 tumor metastases. 5×10^5 B16-C3 cells were injected into the hemispleen of C57BL/6 mice, followed by the intravenously transferred 2.5 $\times 10^6$ activated OT-I CTLs into mice three days later. Time-lapse intravital imaging of the liver metastasis microenvironment was performed one day after OT-I CTLs transfer. Living tumor cells appear in red, apoptotic tumor cells appear in green, and CTLs appear in blue. Scale bar: 40 μm. Total duration = 30 min.

Video 4. The entire process of CTLs killing liver metastasis tumor cells. 5×10^5 B16-C3-OVA cells were injected into the hemispleen of C57BL/6 mice, followed by the intravenously transferred 2.5×10^6 activated OT-I CTLs into mice three days later. Time-lapse intravital imaging of the liver metastasis microenvironment was performed one day after OT-I CTLs transfer. Living tumor cells appear in red, apoptotic tumor cells appear in green, and CTLs appear in blue. Scale bar: 10 μm. Total duration = 150 min.

Video 5. Example of six CTLs (magenta, green, cyan, yellow, red, and gray tracks) that were in contact with one tumor cell and then induced its apoptosis. 5×10^5 B16-C3-OVA cells were injected into the hemispleen of C57BL/6 mice, followed by the intravenously transferred 2.5×10^6 activated OT-I CTLs into mice three days later. Time-lapse intravital imaging of the liver metastasis microenvironment was taken one day after OT-I CTLs transfer. Living tumor cells appear in red, apoptotic tumor cells appear in green, and CTLs appear in blue. Scale bar: 5 μm. Total duration = 90 min.

Video 6. The entire process of CTLs killing tumor cells that were inoculated into the liver *in situ*. 5×10^5 B16-C3-OVA cells were injected into the superficial zones of the livers of C57BL/6 mice, followed by the intravenously transferred 2.5×10^6 activated OT-I CTLs into mice three days later. Time-lapse intravital imaging of the liver metastasis microenvironment was taken one day after OT-I CTLs transfer. Living tumor cells appear in red, apoptotic tumor cells appear in green, and CTLs appear in blue. Scale bar: 10 μm. Total duration = 130 min.

Video 7. The entire process of CTLs killing tumor cells that were inoculated into spleen *in situ*. 5×10^5 B16-C3-OVA cells were injected into the superficial zones of the spleens of C57BL/6 mice, followed by the intravenously transferred 2.5×10^6 activated OT-I CTLs into mice three days later. Time-lapse intravital imaging of the liver metastasis microenvironment was taken one day after OT-I CTLs transfer. Living tumor cells appear in red, apoptotic tumor cells appear in green, and CTLs appear in blue. Scale bar: 10 μm. Total duration = 150 min.

Video 8. Tumor cells with spontaneous short-term calcium influxes in the liver. 5×10^5 B16 cells that expressed Twitch2B calcium sensor and OVA protein were injected into the hemispleens of C57BL/6

mice. Time-lapse intravital imaging of the liver metastasis microenvironment was performed four days after tumor cells inoculation. The orange indicates low calcium signal, the red represents high calcium signal, and blue denotes CTLs. Scale bar: 20 μm. Total duration = 20 min.

Video 9. CTLs in contact with liver metastasis tumor cells, causing prolonged calcium influx to tumor cells. 5×10^5 B16 cells that expressed Twitch2B calcium sensor and OVA protein were injected into the hemispleens of C57BL/6 mice, followed by the intravenously transferred 2.5×10^6 activated OT-I CTLs into mice three days later. Time-lapse intravital imaging of the liver metastasis microenvironment was performed one day after OT-I CTLs transfer. The orange indicates low calcium signal, the red represents high calcium signaling, and blue denotes CTLs. Scale bar: 10 μm. Total duration = 110 min.
